# Inactivation of various variant types of SARS-CoV-2 by indoor-light-sensitive TiO_2_-based photocatalyst

**DOI:** 10.1038/s41598-022-09402-7

**Published:** 2022-04-14

**Authors:** Ryuichi Nakano, Akira Yamaguchi, Kayano Sunada, Takeshi Nagai, Akiyo Nakano, Yuki Suzuki, Hisakazu Yano, Hitoshi Ishiguro, Masahiro Miyauchi

**Affiliations:** 1 Department of Microbiology and Infectious Diseases, Nara Medical University, Kashihara, Nara, 634-8521 Japan; 2 Department of Materials Science and Engineering, School of Materials and Chemical Technology, Tokyo Institute of Technology, Meguro, Tokyo, 152-8552 Japan; 3 Kanagawa Institute of Industrial Science and Technology (KISTEC), Kawasaki, Kanagawa 210-0821 Japan

**Keywords:** Photocatalysis, Photocatalysis

## Abstract

Photocatalysts are promising materials for solid-state antiviral coatings to protect against the spread of pandemic coronavirus disease (COVID-19). This paper reports that copper oxide nanoclusters grafted with titanium dioxide (Cu_x_O/TiO_2_) inactivated the severe acute respiratory syndrome coronavirus 2 (SARS-CoV-2) virus, including its Delta variant, even under dark condition, and further inactivated it under illumination with a white fluorescent bulb. To investigate its inactivation mechanism, the denaturation of spike proteins of SARS-CoV-2 was examined by sodium dodecyl sulphate–polyacrylamide gel electrophoresis (SDS-PAGE) and enzyme-linked immunosorbent assay (ELISA). In addition to spike proteins, fragmentation of ribonucleic acids in SARS-CoV-2 was investigated by real-time reverse transcription quantitative polymerase chain reaction (RT-qPCR). As a result, both spike proteins and RNAs in the SARS-CoV-2 virus were damaged by the Cu_x_O/TiO_2_ photocatalyst even under dark condition and were further damaged under white fluorescent bulb illumination. Based on the present antiviral mechanism, the Cu_x_O/TiO_2_ photocatalyst will be effective in inactivating other potential mutant strains of SARS-CoV-2. The Cu_x_O/TiO_2_ photocatalyst can thus be used to reduce the infectious risk of COVID-19 in an indoor environment, where light illumination is turned on during the day and off during the night.

## Introduction

The novel coronavirus disease (COVID-19) has been breaking out since 2019^1,2^. This pandemic scale disease is caused by the infection of the severe acute respiratory syndrome coronavirus 2 (SARS-CoV-2) virus, mainly through airborne transmission of droplets and/or aerosols produced by infected persons^3^. In addition to the risk of person-to-person contact, active the SARS-CoV-2 viruses have also been detected on the surfaces of objects in public places like hospitals^4^. Therefore, antiviral chemicals and/or materials are useful for protecting against the spread of SARS-CoV-2. Indeed, alcohol^5^, hydrogen peroxide^6^, and hypochlorous acid^7^ have been widely used to inactivate bacteria and/or viruses on surfaces of various objects, such as tables, floors, handrails, touch panels/buttons, and furniture. These chemicals inactivate viruses by denaturing their proteins^8^, however, their antiviral effect is not sustained over the long term because of their evaporation and/or dissipation. Conversely, solid-state antiviral compounds are useful because of their robustness and feasibility for use as coating materials^9^. For example, Ito et al.^10^ reported the inactivation of SARS-CoV-2 by cerium molybdates nanoparticles. Among these various solid-state antibacterial and antiviral materials, titanium dioxide (TiO_2_)-based photocatalysts are promising because of their non-toxic, economical (abundant), chemically and/or thermally stable properties^11–13^, and their antiviral effect can be continuously maintained by illuminating ultraviolet (UV) light^14–16^. Photogenerated holes in the valence band of TiO_2_ have strong oxidation power for decomposing organic molecules; thus, viral components such as surface proteins can be oxidised under UV irradiation, resulting in virus inactivation. Recently, TiO_2_ has been reported to inactivate SARS-CoV-2 through photocatalytic process^17,18^; however, TiO_2_ can only function under UV light, which is hardly contained in a normal room lighting apparatus. Because viral infections mainly occur in indoor environments where many people gather, it is necessary to use a visible-light-sensitive antiviral photocatalyst. It is also noted that lighting is usually turned off during the night; thus, the sustained antiviral property of photocatalysts under dark conditions is also an important requirement for its practical use.

In the present study, we focus on the combination of TiO_2_ with copper oxide as an antiviral composite, although both of them are not catalytically active under dark or visible-light irradiation^19–21^. According to the previous experimental studies, both TiO_2_ and CuO do not so inactivate bacteria well under the dark condition^19,20^. Under UV light irradiation, TiO_2_ can inactivate viruses and/or bacteria^20^, whereas CuO is not photocatalytically active because of its shallow valence band^21^. In other words, neither TiO_2_ nor CuO works well under dark and/or visible light irradiation. In the present study, however, there are two key points for the development of an indoor-active antiviral material. First, we combine TiO_2_ with CuO for visible-light sensitivity by the interfacial charge transfer mechanism^22,23^. Second, we introduce Cu(I) species in CuO resulting Cu_x_O nanoclusters to achieve the efficient antiviral property even under dark, since Cu(I) species work as active sites for antibacterial and antiviral functions^20,24,25^. Recently, our group has developed a visible-light-sensitive antiviral/antibacterial photocatalyst based on copper oxide (Cu_x_O, 1 < x < 2) nanocluster-grafted TiO_2_^25–27^. Cu_x_O nanoclusters are composed of a mixed valence number oxide, in which Cu(I) and Cu(II) species are present. The Cu(II) species in Cu_x_O contributes to the visible-light-driven photocatalysis reaction, whereas the Cu(I) species plays a crucial role in denaturing virus proteins, thereby causing their inactivation under dark conditions. However, these previous studies examined their antiviral properties using bacteriophage Qβ without an envelope, which is a different structure from that of the SARS-CoV-2 (with an envelope). It is worthy to investigate the photocatalytic antiviral property against the not only wild-type strain but also various variant types of SARS-CoV-2 with deep antiviral mechanism study. For example, Delta variant of SARS-CoV-2 spreads faster than the wild-type strain^28^, and mutated Omicron variant recently emerges so far with enhanced transmissibility^29,30^.

Herein, we report the inactivation of the SARS-CoV-2 virus by the Cu_x_O/TiO_2_ photocatalyst and even under dark condition; further, its efficient anti-SARS-CoV-2 performance is achieved under a white fluorescent bulb passed through a UV cutoff filter. The Cu_x_O/TiO_2_ photocatalyst inactivated not only the wild-type strain but also the Alpha, Beta, Gamma, and Delta variants. Since the spike proteins play an important role in SARS-CoV-2 infection, the present study carefully examines the denaturation of spike proteins in SARS-CoV-2 virus by sodium dodecyl sulphate–polyacrylamide gel electrophoresis (SDS-PAGE) and enzyme-linked immunosorbent assay (ELISA) analyses^31^. The SARS-CoV-2 virus has an outer envelope, which involves haphazardly arranged spike proteins that are key to fusing with human cells^32^. In addition to spike proteins, ribonucleic acids (RNAs) in viruses are essential for generating their copies in human cells^33^. Thus, the present study also analyses the defragmentation of RNAs in SARS-CoV-2 by the Cu_x_O/TiO_2_ photocatalyst using a real-time reverse transcription quantitative polymerase chain reaction (RT-qPCR) technique. We carefully discuss the mechanism of the inactivation of SARS-CoV-2 by the Cu_x_O/TiO_2_ photocatalyst under dark and light illumination conditions.

## Results

### Characterisation and photocatalytic activity of Cu_x_O/TiO_2_

The Cu_x_O nanoclusters were facilely grafted onto TiO_2_ powder by a simple impregnation method based on a previous report^25^. Briefly, rutile TiO_2_ powder was dispersed in an aqueous solution of copper chloride (CuCl_2_) under stirring at 90 °C, and then sodium hydroxide and glucose were added to reduce Cu(II) species to Cu(I) species. After washing, filtration, and drying, Cu_x_O/TiO_2_ powder was obtained. For antiviral evaluation, Cu_x_O/TiO_2_ powder was coated onto a glass substrate.

**Figure 1:**
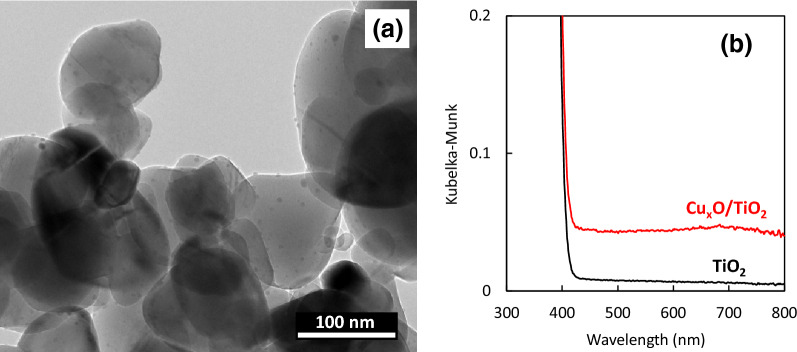
Characterization of Cu_x_O/TiO_2_. (**a**) TEM image of Cu_x_O/TiO_2_ and (**b**) UV–Vis spectra of Cu_x_O/TiO_2_ (red line) and pristine TiO_2_ (black line). Vertical axis of absorption spectra was Kubelka–Munk function, based on the raw data of diffuse reflectance spectra.

Figure 1a shows a transmission electron microscopy (TEM) image of Cu_x_O/TiO_2_. The size of the TiO_2_ powder ranged from approximately 100 to 200 nm, on which small Cu_x_O nanoclusters of a few nanometres in size were grafted. Our energy dispersive X-ray spectrometer (EDS) equipped in a TEM apparatus, scanning electron microscope (SEM) image with its EDS mapping, and X-ray diffraction (XRD) pattern indicated that the grafted small nanoclusters were composed of amorphous copper oxide (see Supplementary Fig. 1–3).

Figure 1b shows the ultraviolet–visible (UV–Vis) absorption spectra of Cu_x_O/TiO_2_ and bare TiO_2_. Bare TiO_2_ absorbs UV light below 400 nm, which is attributed to the band-to-band transition in TiO_2_. On the other hand, Cu_x_O/TiO_2_ exhibits a broad visible light band. The absorption band in the range of 400–500 nm is assigned to interfacial charge transfer (IFCT) from the valence band of TiO_2_ to the unoccupied orbital of Cu(II) species^22,23^, which was confirmed by our in situ electron spin resonance (ESR) analysis under light irradiation (see Supplementary Fig. 4). In addition, the absorption in the range of 500–600 nm originates from the band-to-band transition in Cu_x_O, while the absorption over 650 nm is assigned to the d–d transition of the Cu(II) species^34^. According to a previous report, IFCT transition induced by blue light (around 400–500 nm) irradiation only contributed to the photocatalytic oxidation activity among the observed visible-light absorption bands^25^. Indeed, the present Cu_x_O/TiO_2_ exhibited photocatalytic activity under blue light, while the pristine TiO_2_ did not show any activity under the same blue light (see Supplementary Fig. 5a). The Cu_x_O/TiO_2_ photocatalyst could completely oxidise gaseous 2-propanol molecules into carbon dioxide under visible-light irradiation (Supplementary Fig. 5b). On the basis of its strong oxidising power, the Cu_x_O/TiO_2_ photocatalyst is expected to have high antiviral function. Furthermore, its antiviral activity under dark condition was examined and discussed below.

### Inactivation of SARS-CoV-2

**Figure 2:**
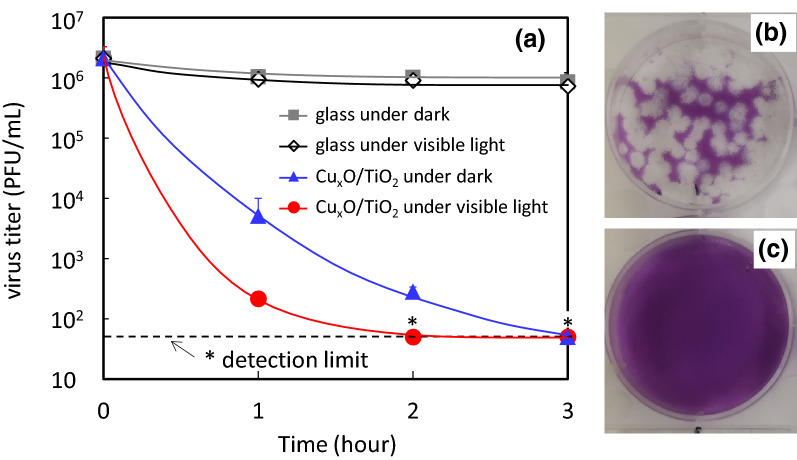
Inactivation of wild-type strain of SARS-CoV-2 by photocatalyst. (**a**) changes of virus titer of SARS-CoV-2 (wild-type strain, 2019-nCoV JPN/TY/WK-521) for each condition, (**b**) photograph of viral plaques infected by SARS-CoV-2 for a bare glass substrate under visible light irradiation, and (**c**) photograph of viral plaques infected by SARS-CoV-2 for Cu_x_O/TiO_2_ photocatalyst under visible light irradiation. White spots in photos indicate the active SARS-CoV-2 virus. Visible light irradiation was conducted using a white fluorescence bulb passed through a UV cutoff filter with the light intensity of 1000 lx. Asterisk marks (*) and dashed line in the panel (**a**) indicate detection limit of the virus.

The photocatalytic antiviral activity of Cu_x_O/TiO_2_ against SARS-CoV-2 virus was evaluated by the method reported in our previous study^15^. Figure 2a shows the inactivation properties of the SARS-CoV-2 virus of wild-type strain under dark and visible light irradiation conditions. Visible light irradiation was conducted using a commercial white fluorescence bulb (see its spectrum in Supplementary Fig. 6), which is usually used as an indoor lighting apparatus. For light irradiation, the UV region was cut off by inserting an optical filter, and the light intensity was set at 1000 lx. We investigated the antiviral properties of the glass substrate without photocatalyst as a control group and found that the virus titer on the glass did not decrease under dark and visible light irradiation conditions. In contrast, the virus titer on the Cu_x_O/TiO_2_ photocatalyst drastically decreased even under dark condition. After 3 h of exposure to Cu_x_O/TiO_2_ in the dark, the virus titer decreased to the detection limit. Furthermore, the antiviral property of the photocatalyst was improved under visible light irradiation, and the virus titer decreased to the detection limit after only 2 h. In other words, four orders of magnitude of the virus were inactivated by the Cu_x_O/TiO_2_ photocatalyst even after 2 h under regular indoor lighting. Figure 2b,c show photographs of SARS-CoV-2 plaques for the glass substrate and Cu_x_O/TiO_2_ photocatalyst under visible light irradiation, where white spots indicate active SARS-CoV-2 viruses. These photos also demonstrate the efficient photocatalytic antiviral properties of our Cu_x_O/TiO_2_ photocatalyst.

**Figure 3:**
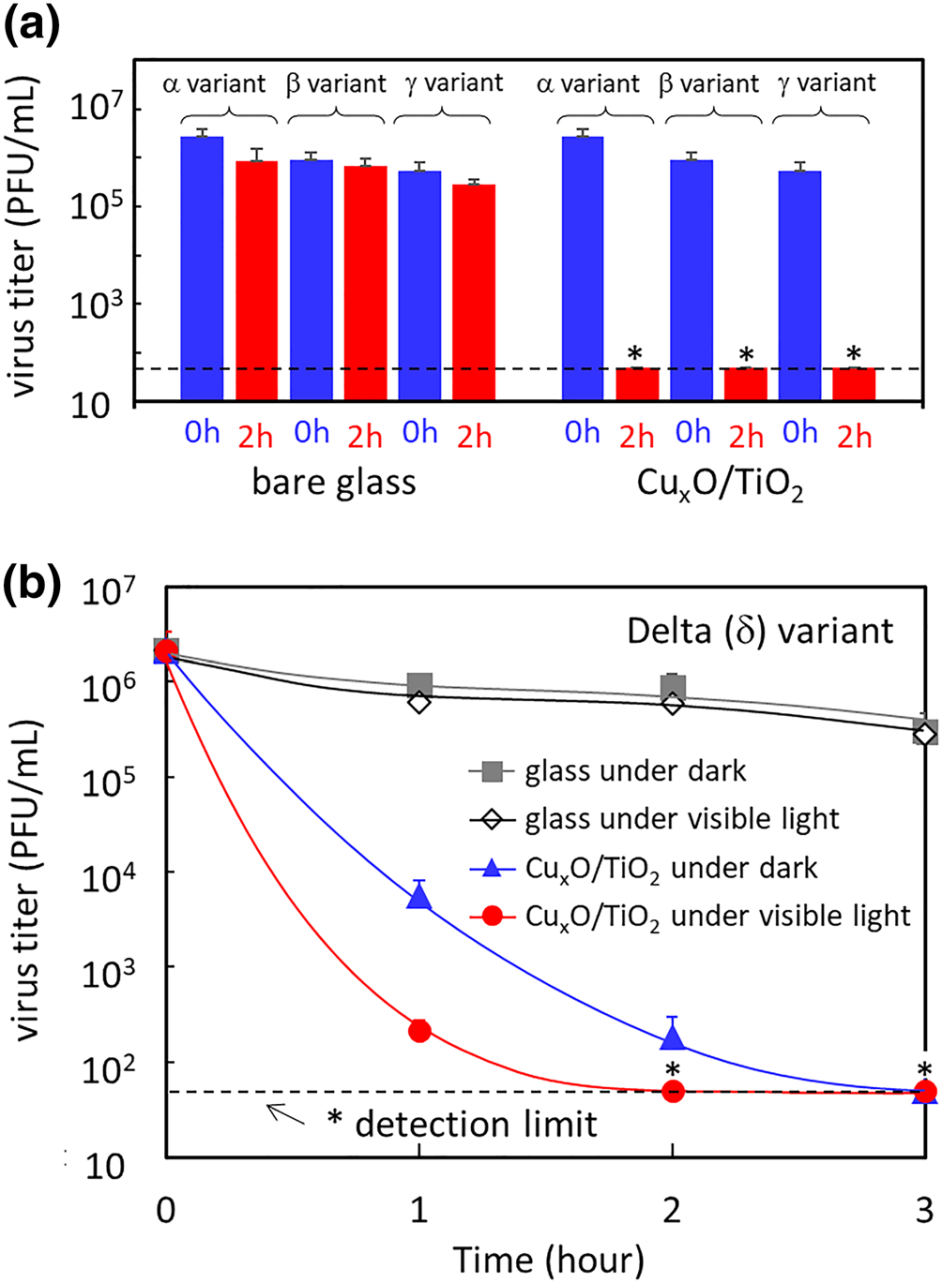
Inactivation of various types of variants. (**a**) changes of virus titer of Alfa (α), Beta (β), and Gamma (γ) variants under 0 and 2 h of visible light irradiation and (**b**) changes of virus titer of Delta (δ) variant. Visible light irradiation was conducted using a white fluorescence bulb passed through a UV cutoff filter with the light intensity of 1000 lx. Asterisk marks (*) and dashed lines in the panels indicate detection limit of viruses.

In addition to the wild-type strain of SARS-CoV-2, we investigated the inactivation properties of Cu_x_O/TiO_2_ versus Alfa (α), Beta (β), Gamma (γ), and Delta (δ) variants. Figure 3a shows the virus titer of Alfa, Beta, and Gamma variants on bare glass and those on the Cu_x_O/TiO_2_ coated sample. The virus titer was examined before light irradiation (dark) and after visible light irradiation for 2 h. Similar to the wild-type strain of SARS-CoV-2 shown in Fig. 2, the Cu_x_O/TiO_2_ disinfected four orders of various variants under visible light irradiation. Figure 3b shows the disinfection of the Delta variant, which is recently causing critical surges in various countries^28^. It is noteworthy that the virus titer of the Delta variant decreased to the detection limit only after 2 h of exposure to the Cu_x_O/TiO_2_ photocatalyst under visible light irradiation and after 3 h of exposure under dark condition.

Previously, Uema et al*.* reported the anti-SARS-CoV-2 properties of a tungsten trioxide (WO_3_)-based photocatalyst^35^, but it did not work well under dark conditions. In this report, the virus titer was reduced by only two orders of magnitude after 3 h under 3000 lx white light irradiation, which was much stronger than the present illumination conditions. TiO_2_ based photocatalyst used in this study has an advantage over these previous studies because of its more efficient anti-SARS-CoV-2 property under visible light irradiation. In addition, our TiO_2_ based material is more chemically stable under alkaline and acidic conditions and is more economical than WO_3_ based photocatalysts. Furthermore, the present Cu_x_O/TiO_2_ exhibits an efficient antiviral activity even in the dark condition, while most of the reported photocatalysts were not functioned under the dark. We previously investigated the antiviral mechanism of Cu_x_O/TiO_2_ using bacteriophage Qβ without an envelope as a virus. It is well understood that the organic substances in bacteriophage Qβ are oxidised to carbon dioxide, causing inactivation of viruses under visible light irradiation. In addition, Cu(I) species in Cu_x_O nanoclusters cause denaturation of protein even in dark condition by the strong adsorption between protein and Cu_x_O^27^. Sunada et al*.*^20,24^ investigated the antiviral mechanism of copper oxides and found that the inactivation property of Cu(I) species was due to its solid-state property toward strong protein adsorption, rather than by the generation of reactive oxygen species or leached copper ions. These previous studies used bacteriophage Qβ without envelope for their mechanism studies; thus, we carefully investigated the denaturation of both spike proteins and ribonucleic acids (RNAs) in SARS-CoV-2 by using SDS-PAGE, ELISA, and RT-qPCR analyses, as presented in the next section.

### Damage of spike proteins and RNAs in SARS-CoV-2 viruses

**Figure 4:**
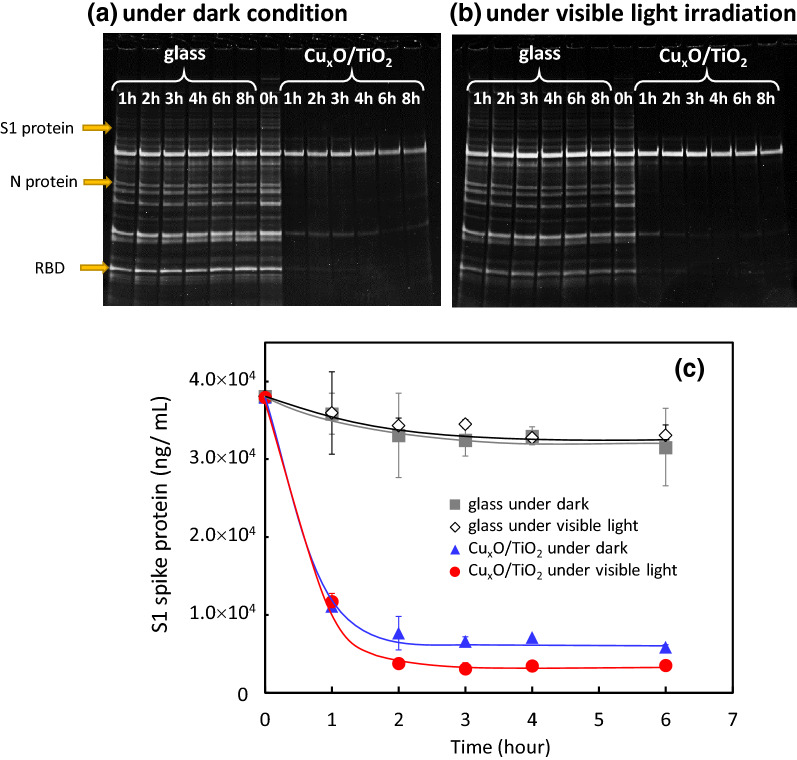
Analysis of SARS-CoV-2 proteins by SDS-PAGE and ELISA. SARS-CoV-2 proteins under (**a**) dark condition and (**b**) visible light irradiation condition for glass substrate and Cu_x_O/TiO_2_ photocatalyst were separated by SDS-PAGE. Lanes 0 h, 1 h, 2 h, 3 h, 4 h, 6 h, and 8 h corresponded to the time after the reaction, respectively. ELISA results (**c**) on SARS-CoV-2 spike S1 protein concentration under dark condition and visible light irradiation condition for glass substrate and Cu_x_O/TiO_2_ photocatalyst. Visible light irradiation was conducted using a white fluorescence bulb passed through a UV cutoff filter with the light intensity of 1000 lx.

**Figure 5:**
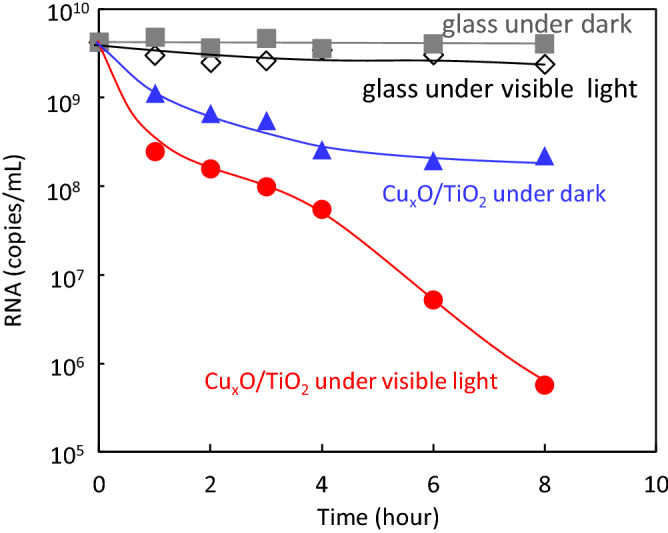
RT-qPCR analysis for SARS-CoV-2 N gene. Changes in RNA copies on glass and Cu_x_O/TiO_2_ photocatalyst under visible light irradiation or dark condition. Visible light irradiation was conducted using a white fluorescence bulb passed through a UV cutoff filter with the light intensity of 1000 lx.

Previous studies have reported that coronavirus-spike (S) glycoproteins promote entry into cells and are the main target of antibodies^31^. Figure 4a,b show the results of SDS-PAGE analysis for Cu_x_O/TiO_2_ and bare glass under dark condition (a) and under visible light irradiation condition (b). It is noteworthy that the band signal of S1 proteins and receptor binding domain (RBD) at S1 proteins were obviously decreased after exposure to the Cu_x_O/TiO_2_ catalyst under dark conditions as well as under visible light irradiation. As RBD recognizes ACE2, a receptor on the surface of host cells^36,37^, the signal disappearance of RBD suggests a decrease in virus infectivity. We also quantitatively evaluated the denaturation of S1 spike proteins by ELISA, and the results are shown in Fig. 4c. The spike proteins were not denatured on a control glass substrate under dark and light illumination conditions. On the other hand, the spike proteins were denatured on the Cu_x_O/TiO_2_ photocatalyst even in the dark, and its denaturation property was further enhanced by visible light irradiation. These results indicate that the Cu_x_O/TiO_2_ photocatalyst denaturalises S1 spike proteins, which play an essential role in entry into lung cells, even under dark conditions as well as under visible light irradiation. Previously, similar denaturation properties to albumin, haemagglutinin, and neuraminidase were observed by Cu(I) species in copper oxides^20,24^. According to these previous studies, the strong antiviral ability of Cu(I) species is owing to its strong adsorption ability towards proteins, rather than the effects of reactive oxygen species or leached copper ions. Therefore, the efficient antiviral activity of Cu_x_O/TiO_2_ photocatalyst even under the dark condition is attributed to its strong adsorption property yielding denaturation of proteins. Protein molecules were further oxidised by the Cu_x_O/TiO_2_ photocatalyst under visible light illumination, because the photogenerated holes in the valence band of TiO_2_ generated through the interfacial charge transfer have strong oxidative power for complete decomposition into carbon dioxide molecules, as shown in the Supplementary Information (Fig. 5).

In addition to spike protein denaturation, we also investigated the fragmentation of RNA in the SARS-CoV-2 virus under exposure to our catalyst. Figure 5 shows the changes in the RNA copies of SARS-CoV-2. Similar to the trends of virus titer and spike proteins shown above, RNA copies were also decreased by exposure to the Cu_x_O/TiO_2_ catalyst, even under dark condition. Furthermore, visible light irradiation of the Cu_x_O/TiO_2_ photocatalyst enhanced the fragmentation of RNAs in SARS-CoV-2. Based on these results, the fragmentation of RNA by the present photocatalyst also contributes to its efficient antiviral activity. The present Cu_x_O/TiO_2_ photocatalyst causes damage to both spike proteins and RNAs in SARS-CoV-2, yielding efficient inactivation under dark condition as well as under indoor light illumination. The change in the number of RNA copies on the Cu_x_O/TiO_2_ photocatalyst under visible light irradiation exhibited a two-step reduction trend (Fig. 5). The initial reduction step (0–4 h) would proceed by similar fragmentation of RNAs under the dark condition. Previously, Qiu et al. investigated the degradation of supercoiled plasmid pBR322 DNA by the present Cu(I) species in Cu_x_O, and they found that the plasmid DNA from the supercoiled to the open circular form was clearly observed by the exposure to Cu(I) species in Cu_x_O even under the dark condition^25^. Therefore, we speculate that the strong adsorption ability of Cu(I) species also causes fragmentation of RNAs even under the dark condition. On the other hand, the second step reduction (4–8 h) would be further driven by the photocatalytic oxidation process, which is able to oxidise organic molecules into water and carbon dioxide molecules. However, we would like to emphasise that complete oxidation is not necessary to inactivate the SARS-CoV-2 virus to the detection limit. As shown in Figs. 2 and 3, an exposure time of 3 h was sufficient for Cu_x_O/TiO_2_ to inactivate SARS-CoV-2 below the detection limit, and even under dark condition. These results indicate that the 3 h exposure to Cu_x_O/TiO_2_ denaturalises spike proteins and also causes fragmentation of RNA, as proven by our SDS-PAGE, ELISA, and RT-qPCR analyses.

**Figure 6:**
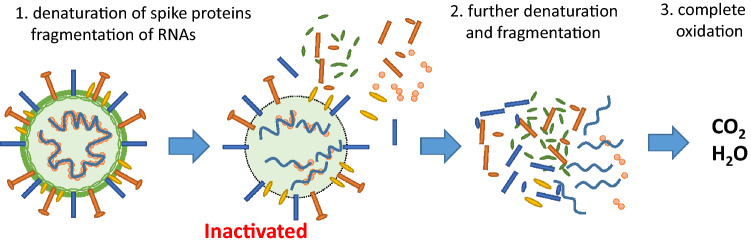
Speculated antiviral mechanism of Cu_x_O/TiO_2_.

Figure 6 shows the antiviral mechanism of the Cu_x_O/TiO_2_ photocatalyst. The Cu(I) species in Cu_x_O denaturalises spike proteins and also causes RNA fragmentation of SARS-CoV-2, even under dark condition, yielding inactivation under the detection limit after only 3 h. Furthermore, light irradiation causes the photocatalytic oxidation of the organic molecules of SARS-CoV-2. Based on this antiviral mechanism, involving denaturation of proteins, fragmentation of RNAs, and oxidation of organic substances by photocatalysis, the Cu_x_O/TiO_2_ photocatalyst is not limited to a specific variant of the virus. It will be effective to inactivate other types of a mutant strain of SARS-CoV-2, such as Omicron strain^29^. It is noted that in the case of vaccines and/or oral drugs, there is a possibility that resistant mutants will emerge in the future. In contrast to vaccines or drugs, the Cu_x_O/TiO_2_ photocatalyst is very useful because it has potential effectiveness against various mutants broadly.

The present study mainly focused on the inactivation of SARS-CoV-2 which consists of an envelope membrane. We also investigated the antiviral properties of feline calicivirus (FCV), which does not have an outer envelope, in contrast to SARS-CoV-2 (see Supplementary Fig. 7). Notably, the present Cu_x_O/TiO_2_ catalyst also inactivated FCVs even under the dark condition, and its performance was further improved under visible-light irradiation. Our previous studies also revealed that the Cu_x_O/TiO_2_ inactivated bacteriophage Qβ, which does not have an envelope membrane^25,27^. These results strongly indicate that the Cu_x_O/TiO_2_ photocatalyst is effective for inactivation of various kinds of viruses by its denaturation and/or strong oxidation ability. Furthermore, Cu_x_O/TiO_2_ exhibited a significant antibacterial effect on *Escherichia coli* and *Staphylococcus aureus*, as well as viruses^25^. Thus, the Cu_x_O/TiO_2_ will be one of the valuable anti-microorganism materials with wide spectrum.

It is important to discuss the toxicity of the present Cu_x_O/TiO_2_ for its practical use. It has been reported the cytotoxic risk of TiO_2_ and CuO nanoparticles towards Zebrafish or colon cells^38–40^. On the other hand, the previous studies directly examined the influence of TiO_2_ and CuO particles on human skin^41,42^. These papers concluded that the TiO_2_ and CuO have extremely low risk to human skin. In the present study, we have conducted the Salmonella reverse mutation assay test (Ames test)^43^ on Cu_x_O/TiO_2_ to discuss its genotoxic risk (Supplementary Material, Table 1 and 2). The test results indicate its extremely low risk. We suppose that the present antiviral particles will be mainly applied as a solid-state coating material on a substrate. Under such applications, concentrated particles are not exposed to human for long term. Therefore, we expect that our antiviral material can be safely used for various coating applications.

## Conclusion

The Cu_x_O/TiO_2_ photocatalyst inactivated SARS-CoV-2 even under dark condition, and its antiviral performance was improved by white light illumination, which is usually used as an indoor light apparatus. Thus, the antiviral function of the Cu_x_O/TiO_2_ photocatalyst can be maintained in an indoor atmosphere, where light illumination is turned on during the day and off during the night without any maintenance, such as spraying of antiviral liquid or wipe-off procedures. The present photocatalyst denaturalises spike proteins and also causes fragmentation of RNAs in the SARS-CoV-2 virus, as proven by SDS-PAGES, ELISA, and RT-qPCR analyses. The Cu_x_O/TiO_2_ photocatalyst has already been commercialised (NAKA CORPORATION, Tokyo Japan) and is expected to be applied to various antiviral industrial items in indoor environments, such as hospitals, airports, metro stations, and schools, as coating materials for air filters, respiratory face masks, and antifungal fabrics to prevent the COVID-19 spread.

## Methods

### Synthesis of Cu_x_O/TiO_2_ powder and film

The Cu_x_O nanoclusters were grafted onto rutile TiO_2_ powder using an impregnation technique. In a typical preparation, one gram of TiO_2_ was dispersed in 10 mL of aqueous CuCl_2_ solution in a vial reactor. The weight fraction of Cu relative to TiO_2_ was 0.25%. During stirring, the temperature of the aqueous solution was maintained at 90 °C for 1 h. Then, sodium hydroxide (NaOH) and glucose were added to the solution (molar ratio of NaOH/glucose/CuCl_2_ = 8/4/1) at the same temperature to graft Cu_x_O nanoclusters onto TiO_2_ particles^25^.

The Cu_x_O/TiO_2_ powder was suspended in 99% ethanol at a concentration of 1 mg/ml. Then, 0.6 ml of the suspension was uniformly loaded onto a soda-lime glass substrate (50 mm × 50 mm) and dried at 100 °C for 15 min. By repeating this operation twice, the glass substrate was thus coated with Cu_x_O/TiO_2_ powder (1.2 mg).

### Characterisation of photocatalyst

Transmission electron microscopy (TEM) images were obtained using a JEM-2100F TEM/STEM (JEOL, Japan) operated at an acceleration electron beam voltage of 200 kV. Field-emission scanning electron microscopy (FE-SEM) images and energy-dispersive X-ray (EDS) analysis were performed using an S4700 (Hitachi High-Tech, Japan). X-ray diffraction (XRD) patterns were recorded using a SmartLab diffractometer (Rigaku, Japan) with Cu Kα radiation (λ = 1.5418 Å). A Si non-reflective plate was used as the substrate. UV–visible (UV–Vis) diffuse reflectance spectra were recorded using a spectrophotometer (V-670, JASCO, Japan) equipped with an integration sphere unit. Optical absorption spectra were obtained using the Kubelka–Munk function^44^ calculated from raw reflection data, where a white BaSO_4_ plate was used as the reflectance standard. Electron spin resonance (ESR) spectra were recorded using an in situ ESR system under light irradiation (EMX Nano, Bruker). For ESR measurements, the photocatalyst powder was placed into a quartz tube filled with nitrogen gas at 90 K with a microwave frequency (X-band) of 9.629 GHz to 9.633 GHz.

### Photocatalytic oxidation activity test

The photocatalytic oxidation activity was evaluated by monitoring the oxidation of gaseous 2-propanol into acetone and carbon dioxide under visible-light irradiation, as the oxidation pathway was well studied in a previous paper^45^. A 100 mg Cu_x_O/TiO_2_ sample was uniformly spread over a glass dish (5.5 cm^2^). Before each photocatalysis test, pre-irradiation was performed overnight in a 500 mL reaction vessel filled with fresh synthetic air to eliminate organic contaminants on the sample surface. Next, the gas inside the reactor was replaced with fresh synthetic air, and 4.1 μmol gaseous 2-propanol was injected into the reactor. Before light irradiation, the system was kept in the dark for 1 h to allow 2-propanol gas to reach absorption equilibrium. Visible light irradiation was performed using a blue light emission diode (LED) with an intensity of 20 mW/cm^2^ measured using a spectro-radiometer (USR-40D, Ushio, Japan), which could drive the interfacial charge transfer (IFCT) in Cu_x_O/TiO_2_. Furthermore, a relatively higher intensity of visible light source (85 mW/cm^2^ by a 150 W Xe lamp with a UV cutoff filter below 420 nm) was also used to investigate whether our photocatalyst could completely oxidise 2-propanol molecules to carbon dioxide. Concentrations of 2-propanol and produced gases of acetone and carbon dioxide were measured using a photoacoustic gas monitor (1412i, INNOVA).

### Virus strain

The SARS-CoV-2 virus reference strains used in this study were the wild-type strain 2019-nCoV JPN/TY/WK-521 and variants of concern (VOCs), including the Alpha variant (B.1.1.7) strain hCoV-19/Japan/QHN001/2020, Beta variant (B.1.351) strain hCoV-19/Japan/TY8-612/2021, Gamma variant (B.1.617.2) strain hCoV-19/Japan/TY7-501/2021, and Delta variant (P.1) strain hCoV-19/Japan/TY11-927-P1/2021, which were isolated and supplied by National Institute of Infectious Disease, Tokyo, Japan, to evaluate the antiviral activity of photocatalysis. These variants were propagated in the African green monkey kidney epithelial cells (Vero E6/TMPRSS2, purchased from Japanese Collection of Research Biosources Cell Bank, National Institute of Biomedical Innovation) which were cultured in Dulbecco’s modified Eagle’s medium (DMEM, Gibco, USA) supplemented with 10% foetal bovine serum (FBS) at 37 °C. Viral titer was determined by the plaque assay technique on confluent layers of Vero E6/TMPRSS2 cell cultures grown in 12-well culture plates as described previously^46,47^. Plaques were quantified and recorded as plaque-forming units (PFU)/mL. All experiments were repeated three separate times, and the average titer was determined; the plaque assay was duplicated in each test. All infection experiments were performed in a biosafety level-3 (BLS-3) laboratory.

### Analysis of Cu_x_O/TiO_2_ inactivation of SARS-CoV-2 virus

The photocatalytic antiviral activity of Cu_x_O/TiO_2_ against SARS-CoV-2 virus was determined according to ISO 18071:2016 (Fine Ceramics [Advanced Ceramics, Advanced Technical Ceramics]—Determination of antiviral activity of semiconducting photocatalytic materials under indoor lighting environment—Test method using bacteriophage Q-beta) and JIS R 1756 with minor modification from our previous study^15^. All experiments were performed in a light-tight box to prevent any influence of indoor light and sunlight. The viral suspension (100 μL) was dropped onto a Cu_x_O/TiO_2_-coated glass plate (50 mm × 50 mm) or non-coated glass as the control and was spread out by covering with an adhesive film (40 mm × 40 mm). The sample was then illuminated with visible light irradiation or under dark condition. Overhead illumination by visible light was provided by using a tubular white fluorescent lamp (FL20SSW/18; Toshiba, Japan) with a UV cutoff filter below 400 nm. The light intensity reaching the surface at the centre of the glass reaction vessel was adjusted to 1000 lx by changing the horizontal distance between the samples and the lamp using a digital illuminance meter IM-5 (TOPCON, Japan). After the reaction time, the virus was collected with 5 mL of phosphate buffered saline (PBS; Sigma-Aldrich Corp., USA) solution, and the virus titer was determined by plaque assay.

### Analysis of SARS-CoV-2 protein on Cu_x_O/TiO_2_ catalyst (SDS-PAGE and ELISA)

After being illuminated with Cu_x_O/TiO_2_-coated glass or control glass for a certain period (0–8 h), all samples were collected and quantitative alterations of the proteins were analysed by sodium dodecyl sulphate–polyacrylamide gel electrophoresis (SDS-PAGE) and enzyme-linked immunosorbent assay (ELISA). For the SDS-PAGE analysis, collected samples were extracted by EzApply (ATTO, Japan) and the protein of the SARS-CoV-2 virus was separated by 10% SDS–polyacrylamide gels as previously described^48^. Protein bands were stained with SYPRO Ruby (Thermo Fisher Scientific, USA) and visualised using a Chemidoc imaging system (BioRad, France).

The quantification of SARS-CoV-2 spike S1 protein of the collected sample was determined using a SARS-CoV-2 Spike S1 Protein ELISA Kit (RK04154; ABclonal, USA), according to the manufacturer's instructions. The calibration standards were assayed at the same time as the samples and allowed the operator to produce a standard curve of optical density versus SARS-CoV-2 spike S1 protein concentration. The concentration of the samples was then determined by comparing the O.D. of the samples to the standard curve. Absorbance was measured at 450 nm using a spectrophotometer. The samples were tested in duplicates.

### Detection of SARS-CoV-2 N gene by RT-qPCR

The quantity of viral RNA was analysed using RT-qPCR. Briefly, RNA was extracted from the samples using the QIAamp Viral RNA Mini Kit (QIAGEN, Hilden, Germany), according to the manufacturer’s instructions. RT-qPCR was performed using the QuantiTect Probe RT-PCR Kit (QIAGEN) on the QuantStudio 5 Real-Time PCR System (ThermoFisher, USA) and the following set of primers/probes specific for the viral N gene: The forward primer, 5’-AAATTTTGGGGACCAGGAAC-3’; the reverse primer, 5’-TGGCAGCTGTGTAGGTCAAC-3’; and the probe, 5’-(FAM) ATGTCGCGCATTGGCATGGA (BHQ)-3′^49^. The cycle threshold (Ct) values of RT-qPCR were converted into viral RNA copy numbers based on a standard curve prepared from tenfold serial dilutions of known copy numbers of SARS-CoV-2 RNA.

## Supplementary Information

Supplementary Information.

## Data Availability

The data that support the findings of this study are available from the article and Supplementary Information files, or from the corresponding authors upon reasonable request.
