# Supplementary material for: Inactivation of various variant types of SARS-CoV-2 by indoor-light-sensitive TiO2-based photocatalyst
Source: Sci Rep. 2022 Apr 14;12:5804. doi: 10.1038/s41598-022-09402-7 (PMC9010443; doi:10.1038/s41598-022-09402-7)
Supplement: Supplementary file 1 — Supplementary Information. [file 41598_2022_9402_MOESM1_ESM.pdf]

Supplementary Information

Inactivation of Various Variant Types of SARS-CoV-2 by Indoor-light-sensitive TiO_2-based Photocatalyst

Ryuichi Nakano,[1*] Akira Yamaguchi,[2] Kayano Sunada,[3] Takeshi Nagai,[3] Akiyo Nakano,[1] Yuki Suzuki,[1] Hisakazu Yano,[1] Hitoshi Ishiguro,[3,*] and Masahiro Miyauchi [2,*]

[1] Department of Microbiology and Infectious Diseases, Nara Medical University, Kashihara, Nara, 634-8521, Japan.

[2] Department of Materials Science and Engineering, School of Materials and Chemical Technology, Tokyo Institute of Technology, Meguro, Tokyo, 152-8552, Japan

[3] Kanagawa Institute of Industrial Science and Technology (KISTEC), Kawasaki, Kanagawa, 210-0821, Japan.

Corresponding authors:
rnakano@naramed-u.ac.jp, pg-ishiguro@newkast.or.jp, mmiyauchi@ceram.titech.ac.jp

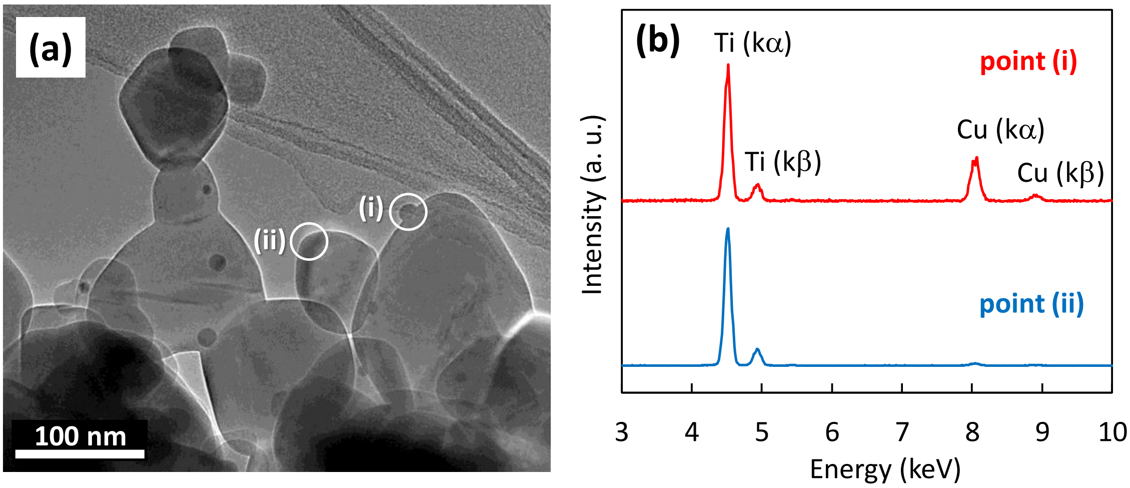

Fig. 1 TEM image and EDS analysis on Cu_xO/TiO_2. (a) TEM image and (b) EDS analysis on the points (i) and (ii) in the panel (a).

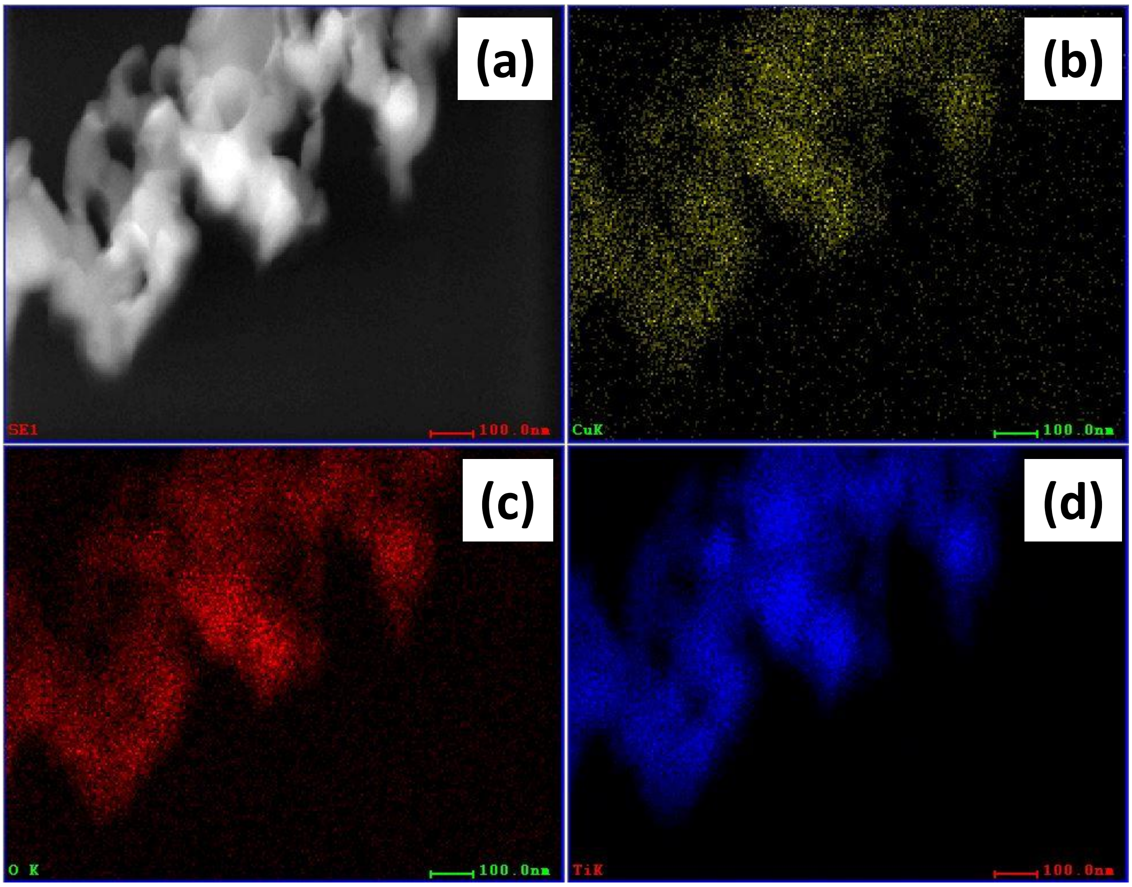

Fig. 2 FE-SEM image and its EDS mapping of Cu_xO/TiO_2. (a) SEM image, (b)-(d) EDS mapping images for copper (yellow), oxygen (red), and titanium (blue), respectively.

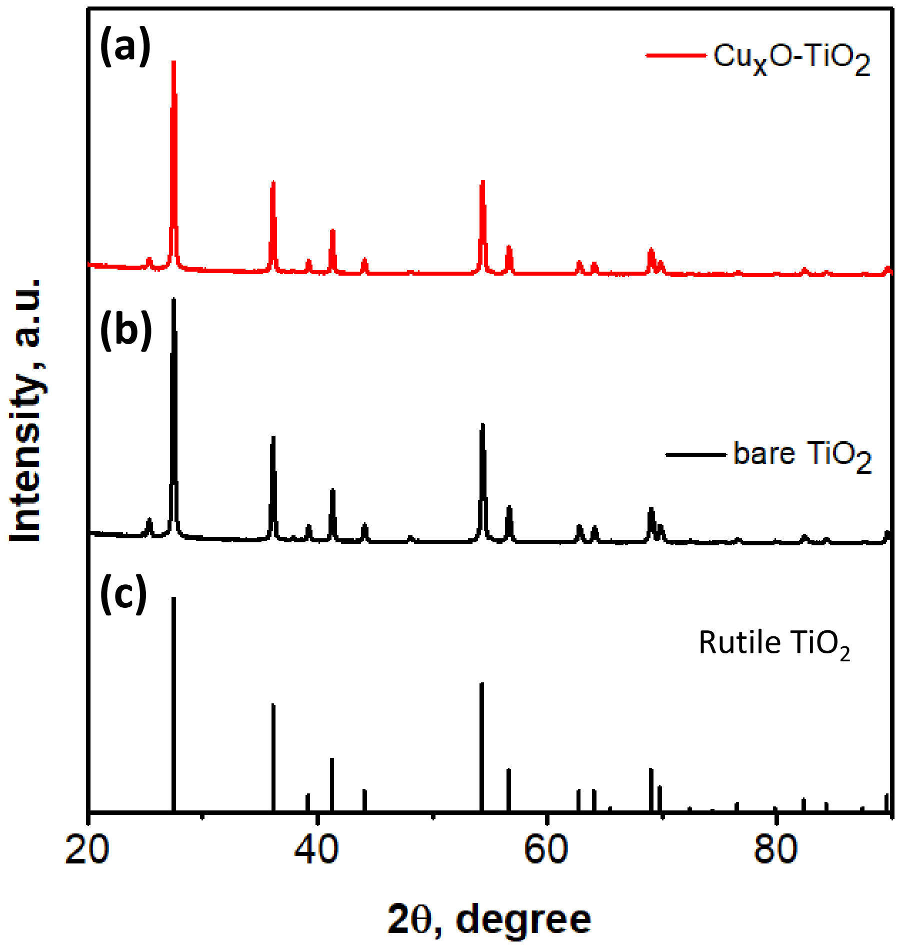

Fig. 3 XRD patterns. (a) Cu_xO/TiO_2, (b) TiO_2, and (c) reported diffraction pattern of TiO_2 in International Centre for Diffraction Data (ICDD File No. 03-065-1118).

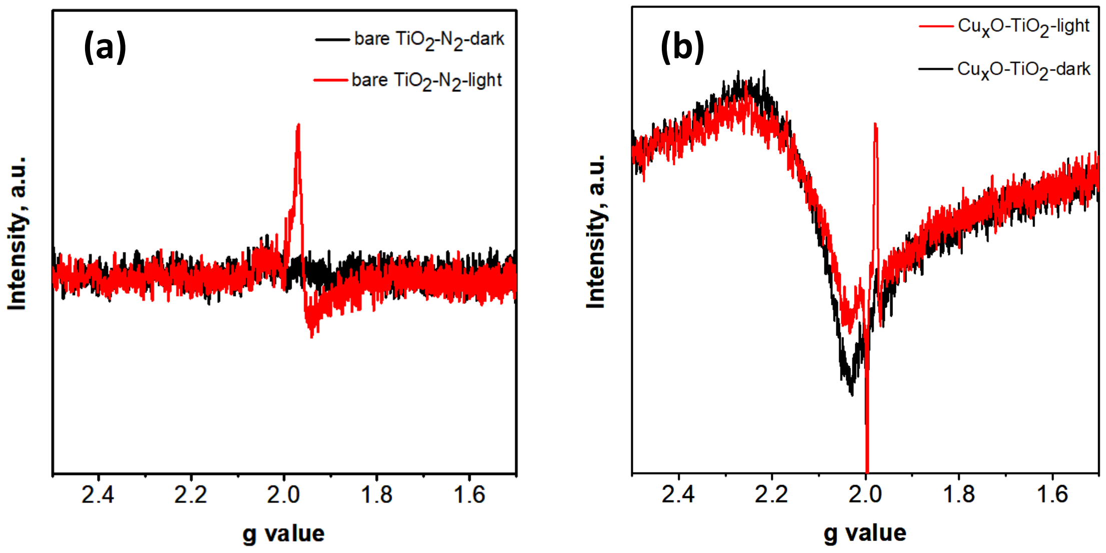

Fig. 4 ESR spectra under dark and visible light irradiation. (a) bare TiO_2 and (b) Cu_xO/TiO_2.

These spectra were recorded under nitrogen condition at 90 K. Sharp signals appeared in both panels at g= 1.98 under visible light irradiation are originated in photo-generated holes in the valence band of TiO_2 (c.f. Nosaka et al. *J. Phys. Chem. C*, 115, 21283, 2011). In the case of Cu_xO/TiO_2, broad signal appeared in the g value from 1.7 to 2.5, which is assigned to the Cu(II) species (c. f. Yin et al. *ACS Nano*, 9, 2111, 2015). Interestingly, the signal of Cu(II) species decreased under visible light irradiation, indicating the formation of Cu(I) species, while holes in the valence band of TiO_2 were generated. These results strongly imply that the IFCT transition proceeds in Cu_xO/TiO_2 *i. e.* electrons in the valence band of TiO_2 are excited to the Cu(II) species under visible light irradiation.

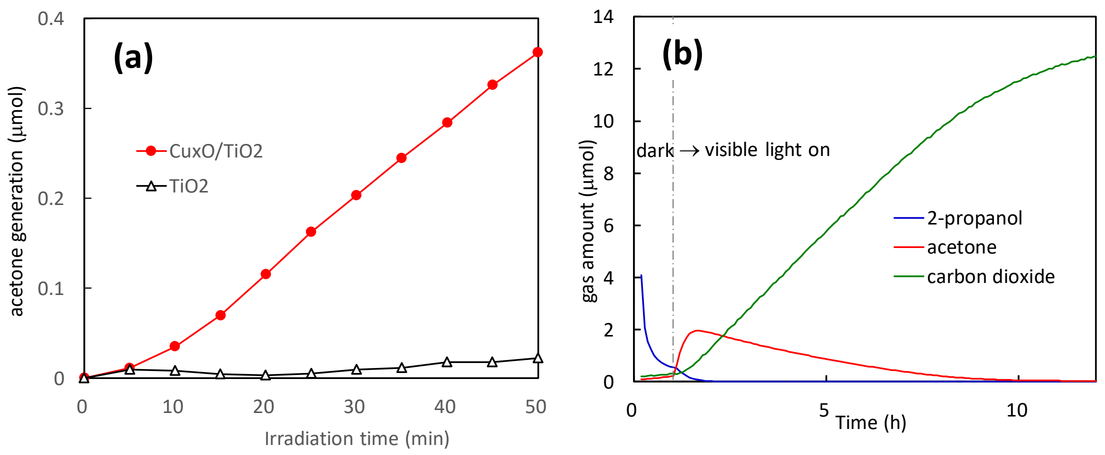

Fig. 5 Photocatalytic oxidation activity of Cu_xO/TiO_2 for the decomposition of gaseous 2-propanol under visible light irradiation. (a) acetone generation from bare TiO_2 and Cu_xO/TiO_2 under blue light emission diode (LED) irradiation, and (b) the time course of 2-propanol, acetone, and carbon dioxide amounts under visible light irradiation using a xenon lamp passed through a UV cutoff filter below 420 nm.

In the case of Cu_xO/TiO_2, 2-propanol was oxidized to acetone under blue LED, whereas the bare TiO_2 did not generate acetone. The panel (b) shows the changes of 2-propanol, acetone, and carbon dioxide amounts under the visible light irradiation over the Cu_xO/TiO_2 photocatalyt. The initial concentration of 2-propanol molecules was 4.1 μmol, which were oxidized to acetone, and further oxidized to carbon dioxide more than 12 μmol. It is noted that a 2-propanol molecule consists of three carbon atoms, thus the present photocatalysis result proves complete oxidation of 2-propanol into carbon dioxide under visible light irradiation over the Cu_xO/TiO_2 photocatalyt.

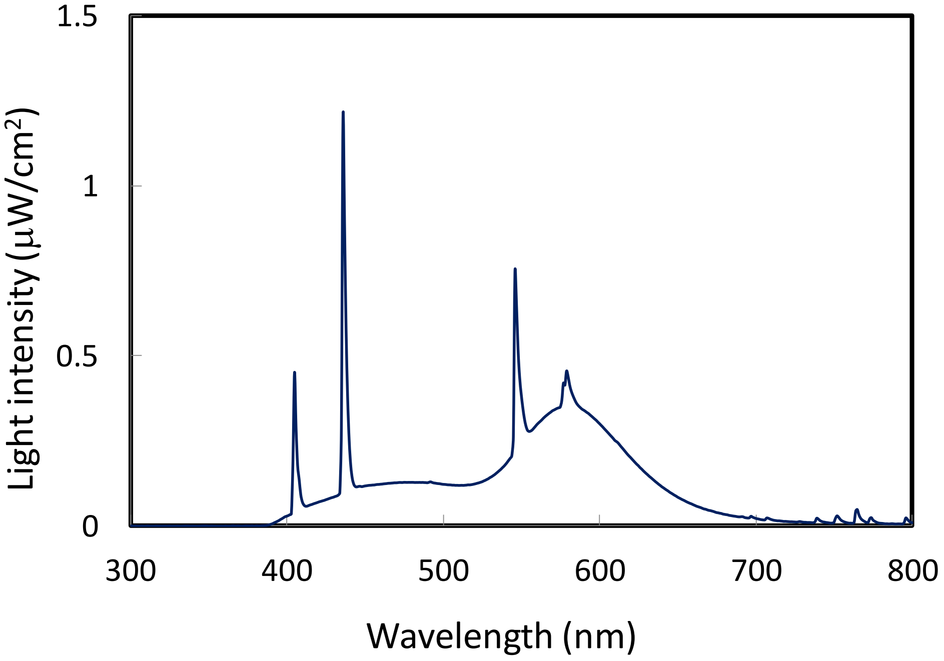

Fig. 6 Spectrum of visible light for the antiviral test. Light irradiation was performed using a white fluorescent light bulb passed through a UV cutoff filter. The light intensity of this spectrum was 1000 lx.

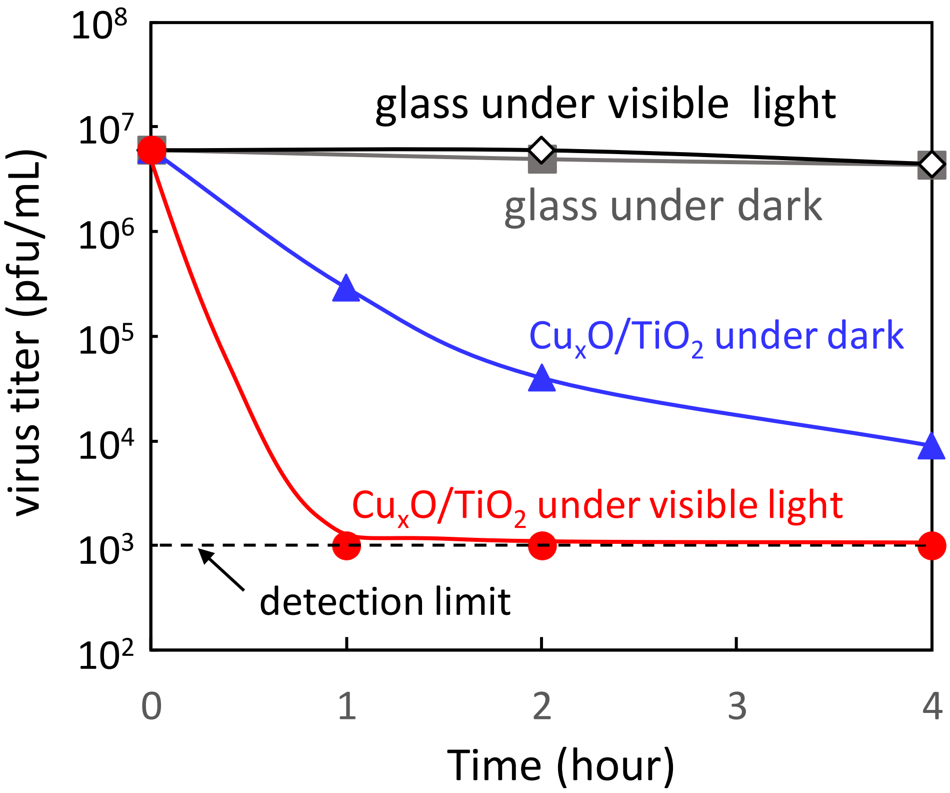

Fig. 7 Inactivation of feline calicivirus (FCV). Changes of virus titer of FCV viruses. Visible light irradiation was conducted using a white fluorescence bulb passed through a UV cutoff filter with the light intensity of 1000 lx.

Ames test

The Ames test [1] has been conducted according to Organization for Economic Cooperation and Development (OECD) Guideline No. 471 (OECD 1987). [2] The tester strains used in the Ames test were *Salmonella typhimurium* TA98, TA100, TA1535, TA1537 and *Escherichia coli* WP2*uvr*A. Five different concentrations of Cu_xO/TiO_2 (78.1, 156, 313, 625, and 1250 μg per plate) were dispersed and shaken at 37 °C for 20 mins with or without S9 mix. The positive controls employed for the assay were 2-(2-Furyl)-3-(5-nitro-2-furyl)acrylamide (AF-2), Sodium azide (SAZ), Benzo[a]pyrene (B[a]P), 2-Methoxy-6-chloro-9-[3-(2-chloroethyl)-aminopropylamino]acridine·2HCl (ICR-191), and 2-Aminoanthracene (2AA), respectively.

The results are shown in Table 1 (without S9 mix) and Table 2 (with S9 mix). The highest concentration of Cu_xO/TiO_2 in this test was 1250 μg per plate, and these conditions did not significantly induce any more colonies than the negative controls. These results reveal that the Cu_xO/TiO_2 exhibited the negative response in all cases suggesting its low genotoxic risk.

References,

1) D. M. Maron, B. N. Ames. Revised methods for the Salmonella mutagenicity test. *Mutat. Res.* **113**, 173–215 (1983).

2) OECD Guideline for Testing of Chemicals: Bacterial Reverse Mutation Test (1987).

Table 1 Ames test results without S9 mix (number of colonies per plate)

strain	TA100	TA1535	WP2*uvr*A	TA98	TA1537
negative control (DMSO)	81<br>91<br>75 (82±8.1)	13<br>11<br>16 (13±2.5)	13<br>11<br>11 (12±1.2)	13<br>22<br>23 (19±5.5)	13<br>11<br>8 (11±2.5)
78.1 μg	80<br>87<br>89 (85±4.7)	6<br>15<br>10 (10±4.5)	15<br>9<br>13 (12±3.1)	19<br>24<br>13 (19±5.5)	6<br>7<br>10 (8±2.1)
156 μg	121<br>94<br>81 (99±20.4)	9<br>12<br>8 (10±2.1)	13<br>10<br>10 (11±1.7)	25<br>20<br>21 (22±2.6)	10<br>6<br>11 (9±2.6)
313 μg	79<br>99<br>89 (89±10.0)	7<br>14<br>7 (9±4.0)	8<br>11<br>11 (10±1.7)	27<br>17<br>21 (22±5.0)	9<br>4<br>9 (7±2.9)
625 μg	106<br>76<br>86 (89±15.3)	5<br>14<br>12 (10±4.7)	18<br>15<br>12 (15±3.0)	15<br>15<br>23 (18±4.6)	6<br>5<br>9 (7±2.1)
1250 μg	83<br>91<br>90 (88±4.4)	10<br>10<br>13 (11±1.7)	11<br>10<br>14 (12±2.1)	22<br>24<br>22 (23±1.2)	7<br>10<br>8 (8±1.5)
positive chemical	**AF-2**	**SAZ**	**AF-2**	**AF-2**	**ICR-191**
dose	0.01μg/plate	0.5μg/plate	0.01μg/plate	0.1μg/plate	1.0μg/plate
	722<br>724<br>721 (722±1.5)	302<br>321<br>325(316±12.3)	80<br>92<br>62 (78±15.1)	420<br>402<br>384(402±18.0)	905<br>819<br>953(892±67.9)

Table 2 Ames test results with S9 mix (number of colonies per plate)

strain	TA100	TA1535	WP2*uvr*A	TA98	TA1537
negative control (DMSO)	104<br>106<br>98 (103±4.2)	18<br>13<br>12 (14±3.2)	15<br>16<br>19 (17±2.1)	25<br>24<br>29 (26±2.6)	17<br>15<br>13 (15±2.0)
78.1 μg	120<br>110<br>103 (111±8.5)	10<br>11<br>12 (11±1.0)	18<br>20<br>21 (20±1.5)	21<br>27<br>24 (24±3.0)	10<br>12<br>12 (11±1.2)
156 μg	106<br>114<br>92(104±11.1)	9<br>12<br>13 (11±2.1)	12<br>8<br>13 (11±2.6)	33<br>34<br>25 (31±4.9)	13<br>13<br>16 (14±1.7)
313 μg	62<br>103<br>71 (79±21.5)	8<br>12<br>13 (11±2.6)	12<br>13<br>15 (13±1.5)	21<br>27<br>20 (23±3.8)	6<br>10<br>10 (9±2.3)
625 μg	92<br>86<br>82 (87±5.0)	13<br>11<br>13 (12±1.2)	13<br>16<br>8 (12±4.0)	33<br>20<br>30 (28±6.8)	7<br>9<br>10 (9±1.5)
1250 μg	79<br>82<br>75 (79±3.5)	11<br>14<br>10 (12±2.1)	8<br>12<br>10 (10±2.0)	35<br>26<br>27 (29±4.9)	10<br>10<br>9 (10±0.6)
positive chemical	**B[a]P**	**2AA**	**2AA**	**B[a]P**	**B[a]P**
dose	5.0μg/plate	2.0μg/plate	10.0μg/plate	5.0μg/plate	5.0μg/plate
	897<br>895<br>937(910±23.7)	247<br>295<br>249(264±27.2)	881<br>892<br>748(840±80.2)	303<br>303<br>337(314±19.6)	91<br>100<br>87(93±6.7)
